# The Seeds of Diversity: Lessons from Tropical Trees

**DOI:** 10.1371/journal.pbio.0040375

**Published:** 2006-10-17

**Authors:** Liza Gross

Understanding the mechanisms that support biodiversity has long been a fundamental problem in ecology. But with species disappearing roughly 1,000 times faster than they did before humans entered the picture, the question is hardly academic.

As biodiversity hotspots, tropical jungles provide a fertile ground for testing theoretical predictions about what drives diversity. Tropical trees of the same species (called conspecifics) often cluster in scales ranging from a few meters to a few hundred meters. Theoretical studies explain why clustering may promote diversity—by separating species and thus reducing competition between them—but evidence supporting different views of what causes clustering has been limited. Studies have established that limited seed dispersal of tropical pioneer trees (the first to colonize a disturbed landscape) in turn limits the spatial distribution of their seeds and seedlings. But without evidence that limited dispersal also affects the spatial distribution of mature trees, the notion that dispersal underlies community structure and biodiversity remains hypothetical.

In a new study, Tristram Seidler and Joshua Plotkin provide that evidence by comparing the dispersal mechanisms and spatial distribution of 561 tropical tree species in a forest reserve in peninsular Malaysia. By demonstrating a strong correlation between the degree of conspecific clustering and the mechanism of dispersal, they show that dispersal characteristics have long-lasting effects on community structure.

Instead of waiting decades for seedlings to mature so they could determine how seed fall affects the spatial distribution of mature trees, Seidler and Plotkin exploited the diversity of dispersal mechanisms across a broad range of species to investigate the relationship. Of 637 tree species within a 50-hectare area, the authors were able to assign dispersal mechanisms to 561 species—based on field data, specimens, and published descriptions of fruit anatomy and morphology. Dispersal mechanisms included ballistic (often described as the “explosive liberation” of seeds), gravity, gyration, wind, and three animal categories based on fruit size. (Smaller passerine songbirds and perching birds, for example, tend to eat and disperse small fruits, while larger birds and mammals tend toward larger fruits.)

To detect patterns in the observed distribution of trees in the study plot, Seidler and Plotkin quantified the degree of spatial aggregation for each species. Based on the observed distribution of conspecific trees, they computed the average spatial cluster size for each species. They found a strong relationship between the cluster size of a species and its dispersal mechanism. Ballistic dispersal produced the most aggregated clusters, followed by gravity, gyration, wind, and animal. (Small-fruited tree species were the most aggregated, and large-fruited trees were the most widely distributed.) These correlations held up after accounting for confounding factors that could arise as a result of evolutionary kinship among species.

**Figure:**
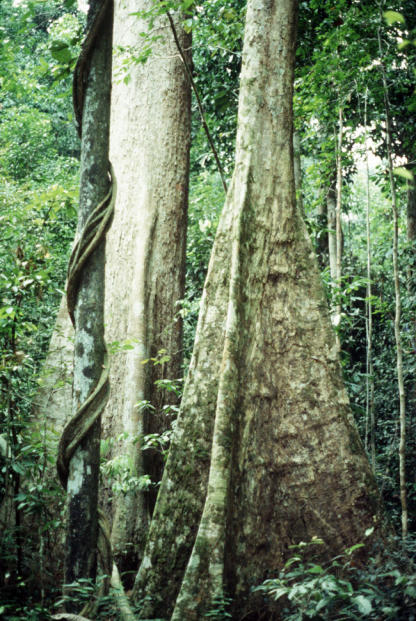
Tree trunks and vines at the Pasoh Forest Reserve, one of the last remaining examples of primary lowland tropical forest in peninsular Malaysia. (Photo: T. Seidler)

To paint a more detailed picture of the relationship between dispersal and spatial pattern, Seidler and Plotkin also quantified aggregation for each species over a range of spatial scales. The relationship between clustering and dispersal mechanism was strongest at smaller scales, while at larger scales (over 200 meters), aggregation did not vary among dispersal mechanisms. The authors found similar patterns in a Panamanian jungle with a markedly different community structure, suggesting that the relationship between dispersal and distribution is not unique to their Malaysian study plot.

These results support several theoretical predictions about the ecological attributes of seed dispersal among tropical trees. The finding that animal-dispersed, small-fruit–bearing trees aggregate more than those producing large fruit supports the hypothesis that larger birds and mammals eat bigger fruits, roam larger territories, and transport seeds farther than smaller animals do. And the tight clustering associated with wind-dispersed species likely results from the forest canopy’s constraining effects on wind currents. The authors caution that their dispersal-mechanism categories are oversimplified and that other factors influence how conspecific trees take root in a particular community. But their results provide direct evidence for theories of how forest communities develop and how variation in dispersal mode promotes diversity—valuable insights for a planet that is losing, or expected to lose, an estimated 0.25% of tropical forest species every year.

